# First report of *Mesocriconema sphaerocephalum* (Taylor, 1936) Loof, 1989 associated with wild grass in Botswana

**DOI:** 10.21307/jofnem-2021-013

**Published:** 2021-03-01

**Authors:** Ebrahim Shokoohi

**Affiliations:** Green Biotechnologies Research Centre of Excellence, University of Limpopo, Private Bag X1106, Sovenga 0727, South Africa

**Keywords:** Botswana, Criconematidae, Molecular, Morphology, Natural area

## Abstract

During a survey on the biodiversity of plant-parasitic nematodes of natural areas in Botswana, *Mesocriconema sphaerocephalum* was discovered around the rhizosphere of the wild grass. The nematodes were extracted using the tray method and then fixed according to the available protocols. The morphological characters fit well with the *M. sphaerocephalum*. Besides, molecular aspects using 18S and 28S rDNA were studied. The phylogenetic analysis of 18S and 28S rDNA placed the examined population with other populations of *M. sphaerocephalum* in a group. According to the knowledge, this is the first report of *M. spaherocephalum* from Botswana.

The genus *Mesocriconema* belongs to the family Criconematidae (Taylor, 1936; Thorne, 1949) comprises over 90 species (Geraert, 2010; Powers et al., 2016). *Mesocriconema xenoplax* (Loof and De Grisse, 1989; Raski, 1952) is the type species distributed worldwide (Geraert, 2010). Members of *Mesocriconema* known as the ring nematode which are ectoparasite and cause yield loss in the high density (Nguyen et al., 2019). However, their ecological behavior has not yet been studied well. During a survey on nematodes of the natural areas of Botswana, *M. sphaerocephalum* (Loof, 1989; Taylor, 1936) was recovered from the wild grass in Botswana. Specimens were collected at the North-West District of Botswana (S 20° 8′24.882″, E 21° 12′45.475″) from the rhizosphere of wild grass plants. To our knowledge, this is the first report of *M. sphaerocephalum* from Botswana.

## Materials and methods

### Nematode extraction, processing, and LM pictures

The specimens were extracted using the tray method and were fixed with a hot 4% formaldehyde solution and transferred to anhydrous glycerin using the De Grisse (1969) method. The classification provided by Geraert (2010) was used for the taxonomical study of *Mesocriconema*. Pictures were taken with a Nikon Eclipse 80i light microscope provided with differential interference contrast optics (DIC) and a Nikon Digital Sight DS-U1 camera (Nikon, Tokyo, Japan). Micrographs were edited using Adobe^®^ Photoshop^®^ CS.

### **D**NA extraction, PCR, and phylogenetic analysis

DNA extraction was done using the Chelex method (Straube and Juen, 2013). Five specimens of each species were hand-picked with a fine tip needle and transferred to a 1.5 ml Eppendorf tube containing 20 μl double distilled water. The nematodes in the tube were crushed with the tip of a fine needle and vortexed. In total, 30 microliters of 5% Chelex^®^ 50 and 2 µL of proteinase K were added to each of the microcentrifuge tubes that contained the crushed nematodes and mixed. These separate microcentrifuge tubes with the nematode lysate were incubated at 56°C for 2 hr and then incubated at 95°C for 10 min to deactivate the proteinase K and finally spin for 2 min at 16,000 rpm (Shokoohi et al., 2020). The supernatant was then extracted from each of the tubes and stored at −20°C. Following this step, the forward and reverse primers, SSU F04 (5′-GCTTGTCTCAAAGATTAAGCC-3′) and SSU R26 (5′-CATTCTTGGCAAATGCTTTCG-3′) (Blaxter et al., 1998); D2A (5″-ACAAGTACCGTGAGGGAAAGTTG-3″) and D3B (5″-TCGGAAGGAACCAGCTACTA-3″) (De Ley et al., 1999), were used in the PCR reactions for partial amplification of the 18S and 28S rDNA region, respectively. PCR was conducted with 8 μl of the DNA template, 12.5 μl of 2X PCR Master Mix Red (Promega, USA) for the Botswanan specimens, 1 μl of each primer (10 pmol μl^−1^), and ddH_2_O for a final volume of 30 μl. The amplification was processed using an Eppendorf master cycler gradient (Eppendorf, Hamburg, Germany), with the following program: initial denaturation for 3 min at 94°C, 37 cycles of denaturation for 45 sec at 94°C; 54°C and 56°C annealing temperatures for 18S and 28S rDNA; extension for 45 sec to 1 min at 72°C, and finally an extension step of 6 min at 72°C followed by a temperature on hold at 4°C. After DNA amplification, 4 μl of product from each tube was loaded on a 1% agarose gel in TBE buffer (40 mM Tris, 40 mM boric acid, and 1 mM EDTA) for evaluation of the DNA bands. The bands were stained with RedGel and visualized and photographed on a UV transilluminator. The amplicons of each gene were stored at −20°C. Finally, the PCR products were purified for sequencing by Inqaba Biotech (South Africa). The ribosomal DNA sequences were analyzed and edited with BioEdit (Hall, 1999) and aligned using CLUSTAL W (Thompson et al., 1994). Phylogenetic trees were generated using the Bayesian inference method as implemented in the program Mr Bayes 3.1.2 (Ronquist and Huelsenbeck, 2003). The HKY + Γ (gamma distribution of rate variation with a proportion of invariable sites) model was selected using jModeltest 2.1.10 (Darriba et al., 2012; Guindon and Gascuel, 2003). Analysis using the HKY + Γ model was initiated with a random starting tree and ran with the Markov chain Monte Carlo (MCMC) for 10^6^ generations for 18S and 28S rDNA. The trees were visualized with the TreeView program. Also, as outgroups, *Basiria gracilis* (DQ328717; MK639375) were selected based on Nguyen et al. (2019). The original partial 18S rDNA and 28S (D2-D3 expansion) sequence of *M. sphaerocephalum* were deposited in GenBank under the accession numbers MW254991-MW254992 (18S rDNA) and MW256823-MW256824 (28S rDNA), respectively.

## Results and discussion

### Morphological characterization

**Figure 1:**
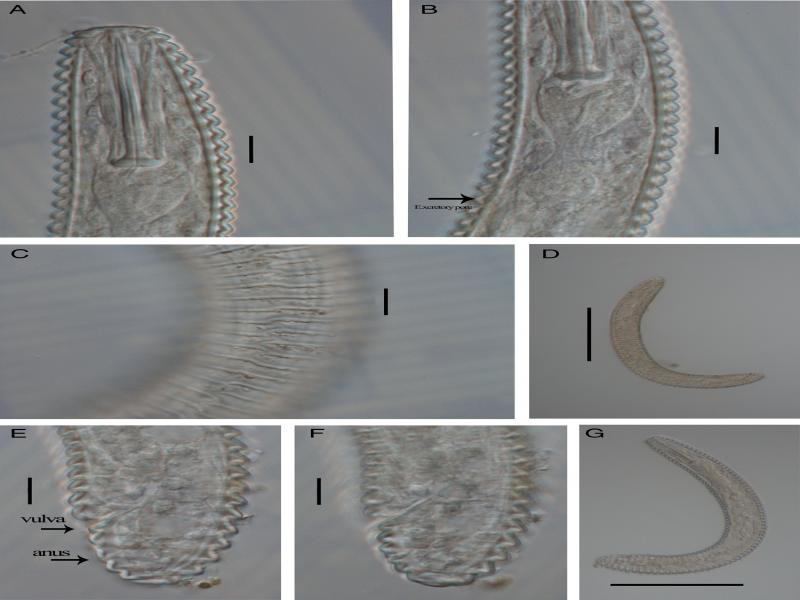
*Mesocriconema sphaerocephalum* (Taylor, 1936; Loof, 1989). (A) Anterior end; (B) pharynx and excretory pore (arrow); (C) lateral field with anastomoses at mid-body; (D, G) entire body; (E, F) posterior end (Scale bar: 10 µm; except for D, G 100 µm).

The morphological and molecular analyses confirmed that the species was *M. sphaerocephalum*. Measurements of *M. sphaerocephalum* in this study are in agreement with the measurement of *M. sphaerocephalum* in Geraert (2010) and Nguyen et al. (2019) (Table 1). Females of *M. sphaerocephalum* are characterized by having body curved ventrally (Fig. 1D, G); lip region with two annuli, slightly flattened labial disc (Fig. 1A); first body annulus much smaller than the second one with smooth edge, sloping posteriorly, (Fig. 1A); cuticle annuli at mid-body 4.2 to 4.6 µm wide. Lateral field with anastomoses, forming zigzag lines (Fig. 1C); stylet robust, knobs 8.6 to 8.9 µm length and 3.2 to 3.4 µm diameter (Fig. 1A, B); vulva located near posterior end; tail rounded in all specimens (Fig. 1E, F). Male not found.

**Table 1.** Measurements of females of *M. sphaerocephalum* from Botswana.

*n*	10 ♀♀
*L*	318 ± 24.8 (275-342)
*a*	9.7 ± 0.4 (9.2-10.2)
*b*	3.4 ± 0.1 (3.2-3.6)
*c*	40.6 ± 4.5 (34.1-44.7)
*c´*	0.5 ± 0.02 (0.4-0.5)
VL/VB	0.6 ± 0.4 (0.7-0.9)
*V*	91 ± 3.5 (88-96)
VL	24 ± 0.5 (23-24)
Stylet	48 ± 2.3 (45-51)
Nerve ring	71 ± 4.9 (67-74)
Pharynx	96 ± 5.7 (87-103)
Excretory pore	102 ± 8.8 (96-115)
Neck base diameter	33 ± 1.4 (31-34)
Vulval-body diameter	28 ± 3.1 (25-32)
Anal body diameter	17 ± 1.8 (16-20)
Tail length	8 ± 0.8 (7-9)
Rst	12 ± 1.0 (11-13)
Rph	22 ± 0.5 (21-22)
Rex	21 ± 1.2 (20-22)
Rv	4.6 ± 0.5 (4-5)
Ran	2.3 ± 0.5 (2-3)
Rvan	2.6 ± 0.5 (2-3)
*R*	66 ± 1.4 (65-67)

**Note:** All measurements are in µm and in form: mean ± SD (range), except for ratio.

The forward SSU F04 and reverse SSU R26 primers of 18S rDNA (Blaxter et al., 1998); forward D2A and the reverse D3B primers of 28S rDNA for *M. sphaerocephalum* isolated 870 and 602 to 630 base pairs long, respectively. The nBlast test of 18S rDNA showed 98% similarity of the test population with the American population of *M. sphaerocephalum* (MF094921). The nBlast of 28S rDNA showed 98% similarity with the Japanese population of *M. sphaerocephalum* (AB933465). Therefore, the molecular results of both 18S and 28S rDNA sequences confirmed our populations as *M. sphaerocephalum*.

**Figure 2:**
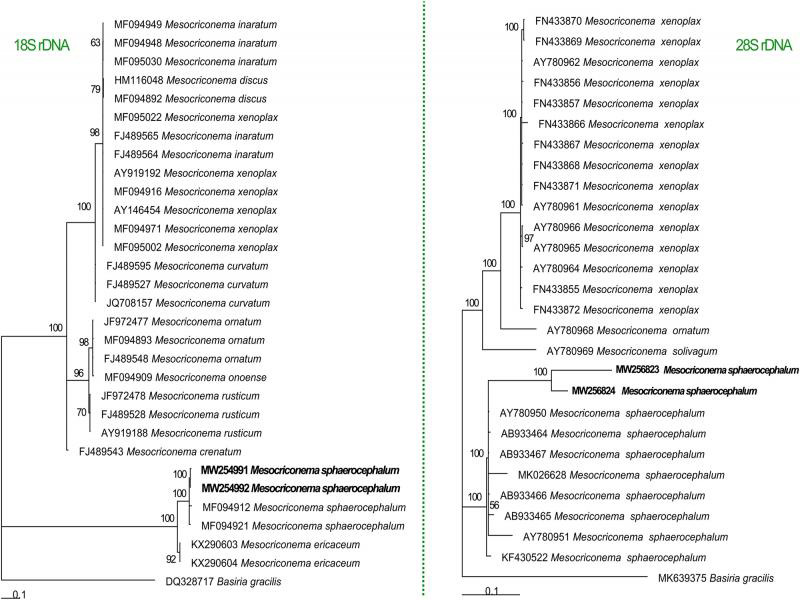
(Left) 18S rDNA and (right) 28S rDNA Bayesian tree inferred from known and newly sequenced *Mesocriconema sphaerocephalum* from Botswana.

The phylogenetic analysis using 18S and 28S rDNA placed the Botswanan *M. sphaerocephalum* population in a clade together with other *M. sphaerocephalum* populations (Fig. 2). The molecular characterization of several sequences of *M. sphaerocephalum* suggested that they formed a monophyletic group. Findings in the current study were in agreement with the phylogenies of *Mesocriconema* species studied using 28S rDNA (Nguyen et al., 2019). Two permanent microscope slides containing 10 females of *M. sphaerocephalum* were deposited in the Nematology collection of the University of Limpopo, South Africa. According to the literature, this is the first record of *M. sphaerocephalum* from Botswana. In conclusion, the ecological behavior of this species needed to be studied to find out the economic importance of the pest.
